# RIPK1 promotes death receptor-independent caspase-8-mediated apoptosis under unresolved ER stress conditions

**DOI:** 10.1038/cddis.2014.523

**Published:** 2014-12-04

**Authors:** Y Estornes, M A Aguileta, C Dubuisson, J De Keyser, V Goossens, K Kersse, A Samali, P Vandenabeele, M J M Bertrand

**Affiliations:** 1 VIB Inflammation Research Center, Technologiepark 927, Zwijnaarde-Ghent 9052, Belgium; 2 Department of Biomedical Molecular Biology, Ghent University, Technologiepark 927, Zwijnaarde-Ghent 9052, Belgium; 3 Apoptosis Research Center, NUI Galway, Galway, Ireland; 4 School of Natural Sciences, NUI Galway, Galway, Ireland

## Abstract

Accumulation of unfolded proteins in the endoplasmic reticulum (ER) causes ER stress and results in the activation of the unfolded protein response (UPR), which aims at restoring ER homeostasis. However, when the stress is too severe the UPR switches from being a pro-survival response to a pro-death one, and the molecular mechanisms underlying ER stress-mediated death have remained incompletely understood. In this study, we identified receptor interacting protein kinase 1 (RIPK1)—a kinase at the crossroad between life and death downstream of various receptors—as a new regulator of ER stress-induced death. We found that Ripk1-deficient MEFs are protected from apoptosis induced by ER stressors, which is reflected by reduced caspase activation and PARP processing. Interestingly, the pro-apoptotic role of Ripk1 is independent of its kinase activity, is not regulated by its cIAP1/2-mediated ubiquitylation, and does not rely on the direct regulation of JNK or CHOP, two reportedly main players in ER stress-induced death. Instead, we found that ER stress-induced apoptosis in these cells relies on death receptor-independent activation of caspase-8, and identified Ripk1 upstream of caspase-8. However, in contrast to RIPK1-dependent apoptosis downstream of TNFR1, we did not find Ripk1 associated with caspase-8 in a death-inducing complex upon unresolved ER stress. Our data rather suggest that RIPK1 indirectly regulates caspase-8 activation, in part *via* interaction with the ER stress sensor inositol-requiring protein 1 (IRE1).

The endoplasmic reticulum (ER) is the main subcellular compartment for protein folding and maturation, and an essential organelle for calcium storage and lipid synthesis. Numerous physiological (e.g., high demand of protein secretion) and pathological (e.g., protein mutation) conditions can alter the proper function of this organelle, leading to accumulation of un- or misfolded proteins in the ER lumen and inducing ER stress. Eukaryotic cells have developed an adaptive molecular response, known as the unfolded protein response (UPR), to sense and adapt to ER stress. In mammalian cells, the UPR emerges from three ER-anchored receptors: inositol-requiring protein 1 (IRE1), protein kinase RNA-like ER kinase (PERK), and activating transcription factor 6 (ATF6). These sensors restore ER homeostasis by activating signaling pathways that increase the folding capacity of the ER, reduce the global synthesis of new proteins, and promote alternative forms of protein degradation. However, in conditions of too severe ER stress, the UPR may fail to restore ER homeostasis and then turns into a toxic signal inducing apoptosis.^1, 2, 3, 4^

ER stress is associated with various human diseases including cancer, neurodegenerative disorders, diabetes, obesity, and inflammatory diseases.^5, 6^ However, although ER stress-induced death is now recognized as an important factor for the development and progression of certain of these diseases, the molecular mechanisms of its induction have remained incompletely understood. Gaining a better understanding of the molecular mechanisms regulating survival and death upon ER stress is therefore of great importance as it may lead to the identification of new therapeutic targets for the treatment of these diseases.

Previous studies have implicated the three branches of the UPR in the death induced by unresolved ER stress, and activation of the intrinsic mitochondrial apoptotic pathway—*via* the regulation of the Bcl-2 family members—is reported as one of the main mechanism for these sensors to promote apoptosis.^2, 3, 7^ Activated PERK and ATF6 have been shown to induce expression of the transcription factor C/EBP-homologous protein (CHOP, also called GADD153), which promotes cell death by regulating the expression of a panel of proteins belonging to the Bcl-2 family such as Bcl-2, Bim, Puma, and Bax.^3, 7^ Moreover, PERK-induced protein synthesis through ATF4- and CHOP-mediated transcription was also reported to generate an ER oxidase 1*α* (ERO1*α*)-dependent oxidative stress leading to IP3-induced calcium release and death.^8, 9, 10^

IRE1 possesses, within its cytoplasmic tail, domains conferring it kinase and endoribonuclease activities. Under ER stress conditions, IRE1 trans-autophosphorylation and oligomerization activates its RNase domain leading to the specific splicing of the transcription factor X-box-binding protein 1 (XBP-1). The spliced form of XBP-1 induces the expression of numerous genes involved in ER homeostasis and survival.^1^ However, under unresolved ER stress conditions, IRE1 may trigger cell death by degrading ER-localized mRNA encoding pro-survival proteins through a process known as regulated IRE1-dependent decay (RIDD).^11, 12, 13, 14^ Recently, RIDD has been reported to contribute to BID-dependent activation of the mitochondrial apoptotic pathway by degrading miRNA repressing expression and activation of caspase-2.^15, 16^ IRE1 is also suggested to promote intrinsic apoptosis by activating c-Jun N-terminal kinase (JNK). Indeed, IRE1 was shown to interact with tumor necrosis factor receptor (TNFR)-associated factor 2 (TRAF2), allowing the downstream activation of the apoptosis signal-regulated kinase 1 (ASK1)/JNK pathway leading to apoptosis.^17, 18, 19, 20^ Sustained JNK activation, as reported to occur during ER stress, is known to trigger apoptosis by regulating the activity of Bcl-2 family members.^21, 22^ Interestingly, activation of JNK by IRE1 was shown to depend on the receptor-interacting serine/threonine protein kinase 1 (RIPK1) *via* TNF-independent interaction of TNFR1 with IRE1 at the ER membrane.^23^ Beside the regulation of the JNK pathway, the IRE1–TRAF2 interaction has also been reported to promote TNFR1-dependent apoptosis by mediating NF-*κ*B-dependent autocrine production of TNF.^24^

RIPK1 is a kinase at the crossroad of life and death, and has a central role in the signaling pathways activated downstream of several TNFR and Toll-like receptor (TLR) family members.^25, 26, 27, 28^ Downstream of TNFR1, RIPK1-dependent cell fate decisions were shown to rely on cellular inhibitor of apoptosis 1 and 2 (cIAP1/2)-dependent ubiquitylation of RIPK1 at the receptor complex (complex I). The ubiquitin chains conjugated to RIPK1 function as a platform for the recruitment and activation of transforming growth factor-beta-activated kinase 1 (TAK1) complex.^29, 30, 31, 32^ Activated TAK1 subsequently activates the mitogen-activated protein kinase (MAPK) and NF-*κ*B pathways, which together drive the transcription of pro-inflammatory and anti-apoptotic genes. In conditions of cIAP1/2 depletion or TAK1 inhibition, RIPK1 dissociates from complex I and mediates, in a kinase-dependent way, the formation of a cytosolic RIPK1/FADD/caspase-8 death complex (complex IIb) that triggers apoptosis.^29, 33, 34, 35^ In condition of caspase inhibition, RIPK1 kinase activity can additionally drive a caspase-independent regulated form of necrosis known as necroptosis.^36^

The fact that RIPK1 is reported to mediate IRE1/TNFR1-dependent JNK activation suggests that RIPK1 could directly contribute to activation of the intrinsic apoptotic machinery resulting from unresolved ER stress.^23^ However, a role for RIPK1 in ER stress-induced apoptosis has not been demonstrated yet. In this study, we show that RIPK1 deficiency protects the cells from ER stress-triggered apoptosis but, surprisingly and in contradiction with previous studies, found that this protection was not the result of a defect of JNK activation or TNFR1 signaling. Instead, we found that RIPK1 promotes death receptor-independent caspase-8 activation, which is required for the downstream activation of caspase-9 and -3 and the induction of cell death. Interestingly, we found that RIPK1 interacts with IRE1, which may initiate part of the apoptotic function of RIPK1.

## Results

### RIPK1 is required for ER stress-induced apoptosis

**Figure 1:**
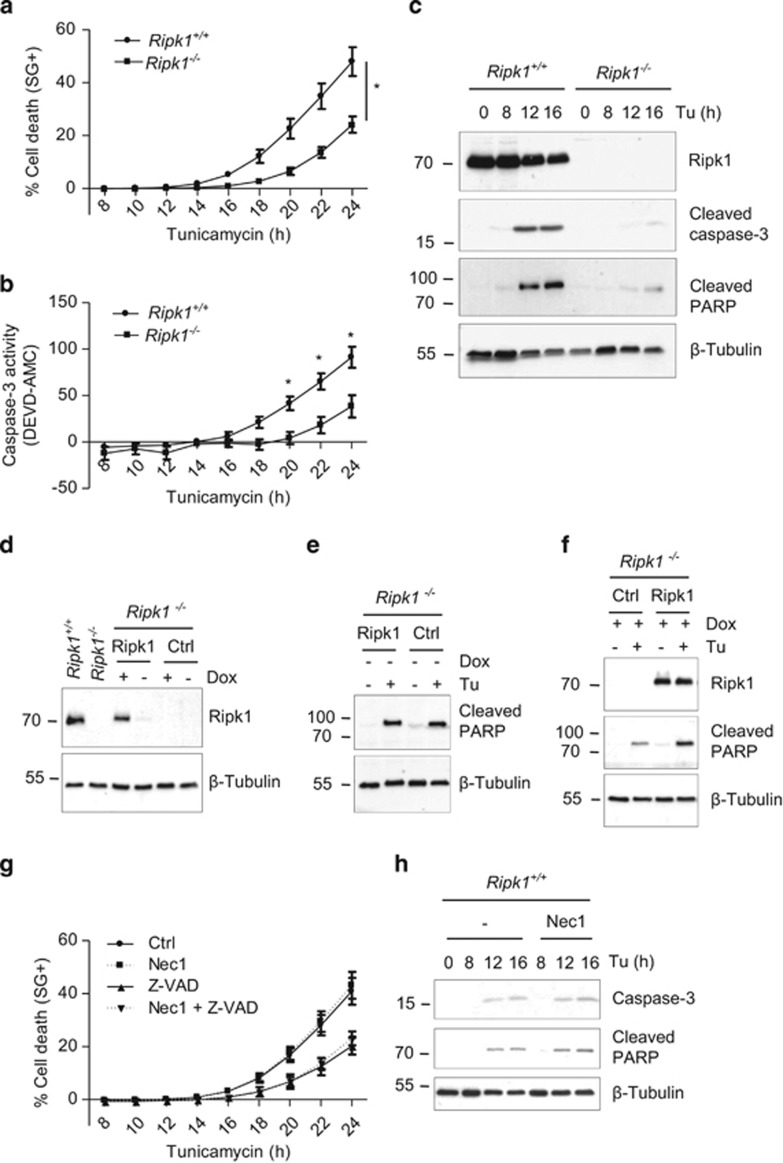
RIPK1 mediates ER stress-triggered apoptosis independently of its kinase activity. (**a** and **b**) *Ripk1*^*+/+*^ and *Ripk1*^*−/−*^ MEFs were stimulated with 1 *μ*g/ml tunicamycin (Tu), and the percentage of cell death (**a**) or caspase-3 like activity (**b**) was measured in function of time using the Fluostar Omega fluorescence plate reader.*P<0.01; error bars represent S.E.M. of six (**a**) and three (**b**) independent experiments. (**c**) *Ripk1*^*+/+*^ and *Ripk1*^*−/−*^ MEFs were incubated with 1 *μ*g/ml Tu for the indicated time, and cell lysates were immunoblotted as indicated. (**d**–**f**) *Ripk1*^*−/−*^ MEFs reconstituted with a doxycycline-inducible Ripk1 coding vector or with an empty vector (Ctrl) were treated (+) or not (−) with doxycycline (Dox) and then exposed (+) or not (−) to 1 *μ*g/ml Tu. The cells were then lysed and immunoblotted as indicated. (**g**) *Ripk1*^*+/+*^ MEFs were incubated for 30 min with necrostatin-1 (Nec1) in the presence or absence of Z-VAD-fmk (Z-VAD), and then stimulated with 1 *μ*g/ml Tu. The percentage of cell death was measured as in (**a**). Error bars represent S.E.M. of three independent experiments. (**h**) *Ripk1*^*+/+*^ MEFs were incubated for 30 min with Nec1 and then stimulated with 1 *μ*g/ml Tu for the indicated time, and the cell lysates were immunoblotted as indicated

RIPK1 has been previously linked to the UPR by regulating IRE1/TNFR1-mediated JNK activation.^23^ To investigate the role of RIPK1 in ER stress-mediated death, we compared the effect of the well-established ER stress inducer tunicamycin on *Ripk1*^*+/+*^ and *Ripk1*^*−/−*^ MEFs. Ripk1-deficient cells exhibited a strong delay in death induced by tunicamycin when compared with wild-type (WT) counterparts, resulting in an ~50% protection after 24 h of treatment (Figure 1a). When treated for a longer period of time, *Ripk1*^*+/+*^ MEFs reached 100% cell death much earlier than *Ripk1*^*−/−*^ MEFs, indicating that the delay in the death observed during the first 24 h was maintained over time (Supplementary Figure S1a). Moreover, cell death induction by two other ER stress inducers, thapsigargin and brefeldin A, was also strongly reduced in Ripk1-deficient cells compared with the WT counterpart (Supplementary Figure S1b and c). These results indicate that Ripk1 deficiency confers a general protection against ER stress-mediated death.

In correlation with the death profiles, caspase-3-like activity was reduced in *Ripk1*^*−/−*^ compared with *Ripk1*^*+/+*^ cells (Figure 1b), and Ripk1 deficiency led to a great reduction in tunicamycin-induced caspase-3 and PARP processing (Figure 1c), indicating that Ripk1 mediates apoptosis triggered by ER stress. Accordingly, caspase processing was also reduced in *Ripk1*^*−/−*^ cells stimulated with thapsigargin (Supplementary Figure S1d). This defect in cell death induction was not the result of an intrinsic defect of the cells, since *Ripk1*^*−/−*^ cells were sensitized to TNF-induced death (Supplementary Figure S2), as previously described.^37^

To further demonstrate the role of RIPK1 in ER stress-induced apoptosis, we stably reconstituted *Ripk1*^*−/−*^ cells with WT Ripk1, or with an empty control vector, using a doxycyclin-inducible system. Doxycycline treatment of reconstituted *Ripk1*^*−/−*^ cells led to a level of Ripk1 expression comparable to the endogenous one (Figure 1d). In addition, in the absence of doxycycline, tunicamycin-induced caspase-3 activation (monitored by PARP processing) was similar in cell reconstituted with Ripk1 or with the control vector (Figure 1e). By contrast, the doxycycline-induced re-expression of Ripk1 greatly enhanced PARP processing upon tunicamycin treatment (Figure 1f), confirming a role for Ripk1 in ER stress-mediated apoptosis.

To investigate whether the pro-apoptotic role of RIPK1 during ER stress requires its enzymatic activity, we tested the effect of necrostatin-1 (Nec1), an inhibitor of RIPK1 kinase activity.^38^ We found that Nec1 had no effect on tunicamycin-induced death or caspase-3 processing (Figures 1g and h). Of note, blockade of caspases with the broad-spectrum caspase inhibitor Z-VAD only partly protected the cells from tunicamycin-mediated death (Figure 1g), indicating that caspase-independent death also occurs following ER stress, and/or that caspase inhibition induces a switch to a non-apoptotic cell death modality—possibly necroptosis. Nevertheless, combining Nec1 with Z-VAD did not enhance the protection given by Z-VAD alone, implying that the remaining death is not Ripk1 kinase-dependent necroptosis.

cIAP1/2 are direct ubiquitin ligases for RIPK1, and cIAP1/2 depletion has been reported to sensitize cells to RIPK1-dependent apoptosis downstream of TNFRs.^28, 29, 34, 35, 39^ In addition, it has been shown that upregulation of cIAP1/2 in response to ER stress has a survival role.^40, 41, 42, 43^ However, stimulation of the cells with the Smac mimetic BV6, a compound that induces proteasomal degradation of cIAP1/2,^44^ did not sensitize the cells to tunicamycin-induced caspase-3 processing or cell death (Supplementary Figure S3a and b). Of note, tunicamycin treatment did not induce notable increase of cIAP1 expression in *Ripk1*^*+/+*^ MEFs (Supplementary Figure S3c).

Taken together, these results demonstrate that RIPK1 mediates ER stress-induced apoptosis independently of its kinase activity, and that cIAP1/2 are not involved in this process.

### The pro-apoptotic function of RIPK1 is independent of JNK or CHOP regulation

**Figure 2:**
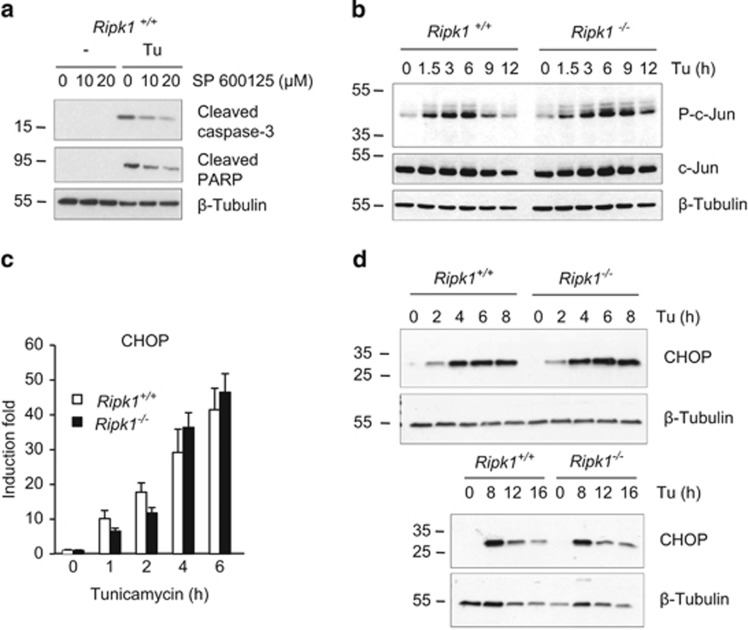
RIPK1 does not regulate JNK or CHOP during ER stress. (**a**) *Ripk1*^*+/+*^ MEFs were incubated for 30 min with 10 or 20 *μ*M JNK inhibitor SP 600125 and then exposed to 1 *μ*g/ml tunicamycin (Tu) for 12 h. The cells were then lysed and immunoblotted as indicated. (**b**–**d**) *Ripk1*^*+/+*^ and *Ripk1*^*−/−*^ MEFs were exposed to 1 *μ*g/ml Tu for the indicated time and cell lysates were either immunoblotted as indicated (**b** and **d**), or used to measure the expression of CHOP mRNA by RT-qPCR (**c**). Error bars indicate the standard deviation from triplicate samples. The result is representative of two independent experiments

RIPK1 has been reported to mediate JNK activation during ER stress.^23^ Knowing the implication of the JNK pathway in ER stress-induced apoptosis,^3, 18, 20, 22^ we logically hypothesized that the protection of the *Ripk1*^*−/−*^ cells to tunicamycin-induced apoptosis was a result of defective JNK activation. To test this hypothesis, we first analyzed the role of the JNK pathway in our cellular system by using the JNK inhibitor SP600125. This inhibitor was functional as it completely blocked the phosphorylation of c-Jun—a well-known substrate of JNK^45^—following stimulation with TNF or IL-1*β* (Supplementary Figure S4). Pretreatment of *Ripk1*^+/+^ cells with SP600125 decreased, in a dose-dependent manner, the processing of caspase-3 and PARP triggered by tunicamycin (Figure 2a). However, and unexpectedly, Ripk1 deficiency did not alter activation of JNK—monitored by the phosphorylation of c-Jun—in cells stimulated with tunicamycin (Figure 2b). Hence, even though we confirmed the link between JNK activation and ER stress-induced apoptosis,^2, 3^ our results argue against a role of RIPK1 in JNK activation. Our data therefore indicate that the pro-apoptotic function of RIPK1 relies on the activation of another pro-death pathway.

Several *in vitro* and *in vivo* studies have highlighted the importance of CHOP for apoptosis induction during ER stress.^2, 3^ We therefore compared the induction of CHOP in *Ripk1*^*+/+*^*versus Ripk1*^*−/−*^ cells. RT-qPCR and immunoblot analysis revealed that neither the mRNA nor the protein levels of CHOP were affected by Ripk1 deficiency in response to tunicamycin or thapsigargin treatment (Figures 2c and d; Supplementary Figure S5), indicating that the pro-apoptotic role of Ripk1 is independent of CHOP regulation. In addition, Ripk1 deficiency did not alter the IRE1-dependent, spliced XBP-1-mediated survival pathway, since the kinetics of Xbp-1 mRNA splicing and translation between Ripk1-deficient and -proficient cells were identical (Supplementary Figure S6).

Taken together, these data demonstrate that the pro-apoptotic function of RIPK1 does not involve JNK activation, CHOP induction, or inhibition of XBP-1 splicing.

### RIPK1 acts upstream of caspase-8 activation during unresolved ER stress

**Figure 3:**
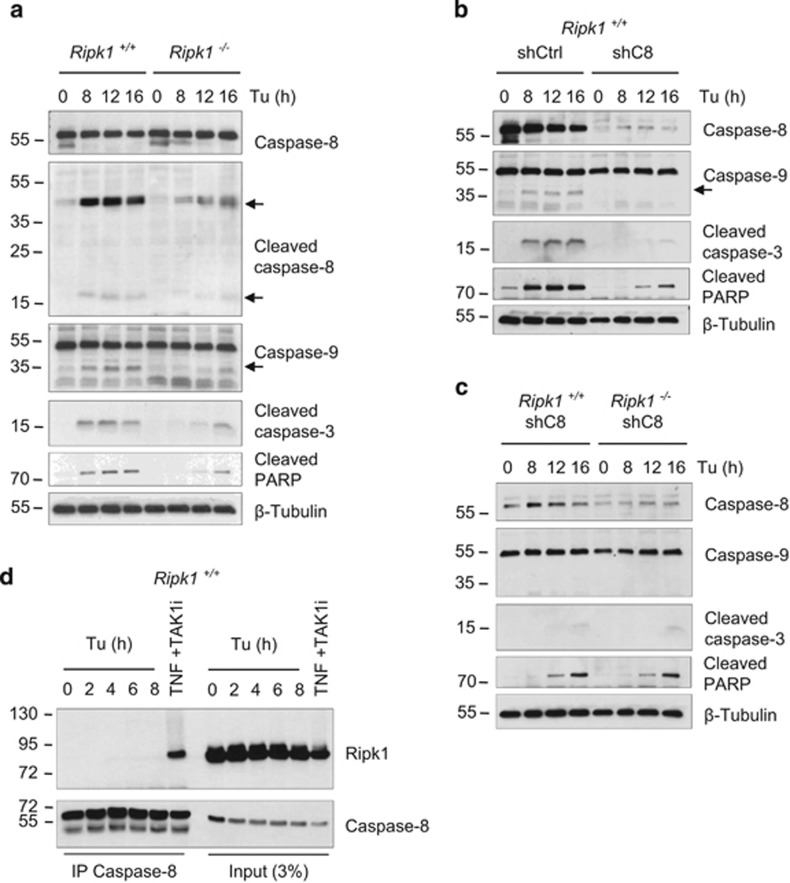
Ripk1 acts upstream of caspase-8 during ER stress-mediated death. (**a**) *Ripk1*^*+/+*^ and *Ripk1*^*−/−*^ MEFs were exposed to 1 *μ*g/ml tunicamycin (Tu) for the indicated time, and then lysed and immunoblotted as indicated. (**b** and **c**) *Ripk1*^*+/+*^ and *Ripk1*^*−/−*^ MEF transduced with a control shRNA (shCtrl) or with shRNA targeting caspase-8 (shC8) were treated with 1 *μ*g/ml Tu, and then lysed and immunblotted as indicated. Arrows indicated cleaved fragments of proteins. (**d**) *Ripk1*^*+/+*^ MEFs were stimulated with 1 *μ*g/ml Tu for the indicated time, or incubated for 30 min with a TAK1 inhibitor (TAK1i) before stimulation with TNF for 2 h. Caspase-8 immunoprecipitates (IP) and cell lysates (Input 3%) were analyzed by immunoblot (IB) as indicated

ER stress is widely reported to kill by activating the intrinsic apoptotic pathway,^3^ and caspase-2 has been suggested to act as the initiator caspase activating the mitochondrial pathway.^46, 47^ The analysis of our cell lysates did not allow us to detect caspase-2 processing (data not shown). Instead, on top of caspase-9 and -3 cleavage, we detected clear evidence of caspase-8 processing, an initiator caspase typically activated by death receptors in the extrinsic apoptotic pathway (Figure 3a). Importantly, as previously observed for caspase-3 and PARP, the cleavage of both caspase-8 and caspase-9 was strongly decreased in the absence of Ripk1, therefore placing Ripk1 upstream of these caspases in the death pathway (Figure 3a). Of note, knockdown of caspase-8 by stable shRNA transfection prevented caspase-9, -3 and PARP processing, revealing a role of caspase-8 upstream of the apoptosome (Figure 3b). In agreement, caspase-8-deficient JA3 Jurkat cells were protected against tunicamycin- and thapsigargin-induced caspase-3 processing and death (Supplementary Figure S7). Finally, in line with the hypothesis that RIPK1 and caspase-8 act in the same apoptotic pathway, the double invalidation of Ripk1 and caspase-8 in MEFs did not further improve the protection given by caspase-8 repression alone (Figure 3c). These results therefore reveal that RIPK1-dependent caspase-8 processing is crucial for ER stress-mediated apoptosis, and suggest that caspase-8 is the primarily initiator caspase activated under ER stress conditions.

To further investigate the role of RIPK1 in the control of caspase-8 activation, we examined whether Ripk1 and caspase-8 interacted in response to ER stress in MEFs. As previously reported,^33^ an interaction between caspase-8 and RIPK1 was detected when the cells were stimulated with TNF in the presence of TAK1 inhibitor (Figure 3d), validating the efficiency of our caspase-8 immunoprecipitations. However, we did not detect the presence of Ripk1 in caspase-8 immunoprecipitates when the cells were treated with tunicamycin (Figure 3d). These results therefore suggest that the formation of an RIPK1-caspase-8 containing death complex in response to ER stress is unlikely and that RIPK1 indirectly regulates caspase-8 activation.

### Caspase-8 activation occurs independently of the classical death receptor signaling

**Figure 4:**
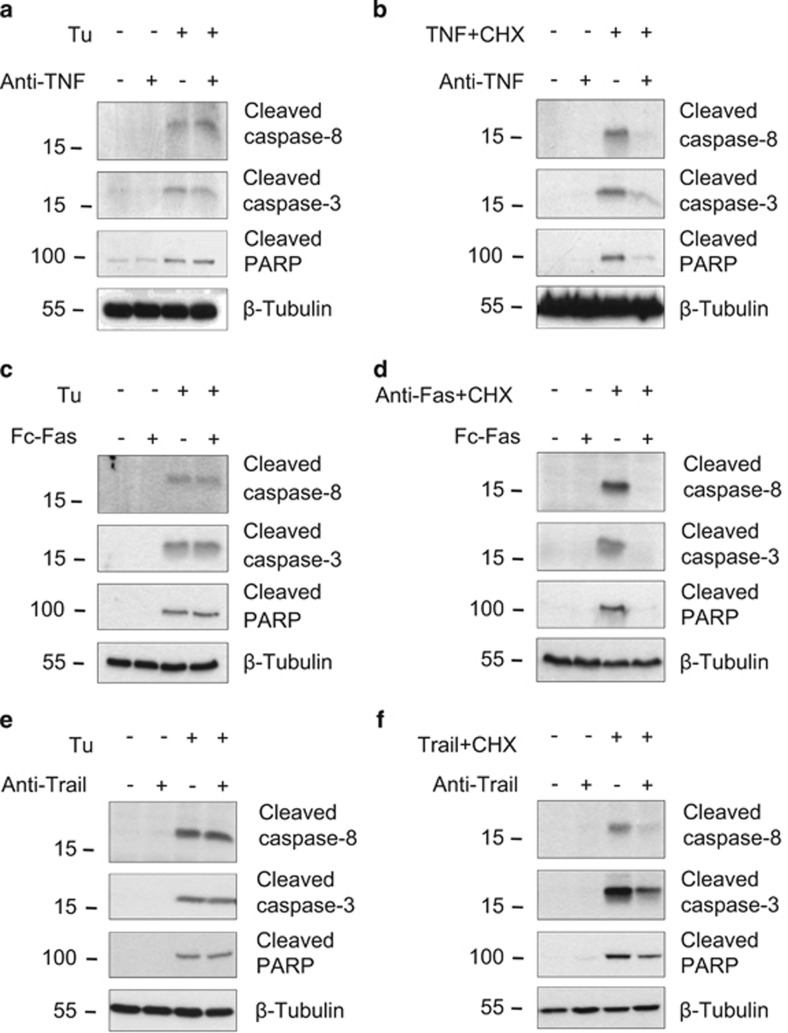
ER stress-induced caspase-8 activation occurs independently of autocrine production of TNF, Fas ligand, or TRAIL. (**a**–**f**) *Ripk1*^*+/+*^ MEFs were incubated for 30 min with 10 *μ*g/ml anti-TNF (**a** and **b**) or anti-TRAIL (**e** and **f**) blocking antibodies, or recombinant mouse Fas:Fc chimera (**c** and **d**), and then stimulated with 1 *μ*g/ml tunicamycin (Tu) (**a**, **c**, and **e**), or with TNF (**b**), anti-Fas antibody (**d**), or TRAIL (**f**) in combination with cycloheximide (CHX). The cells were then lysed and immunoblotted as indicated

**Figure 5:**
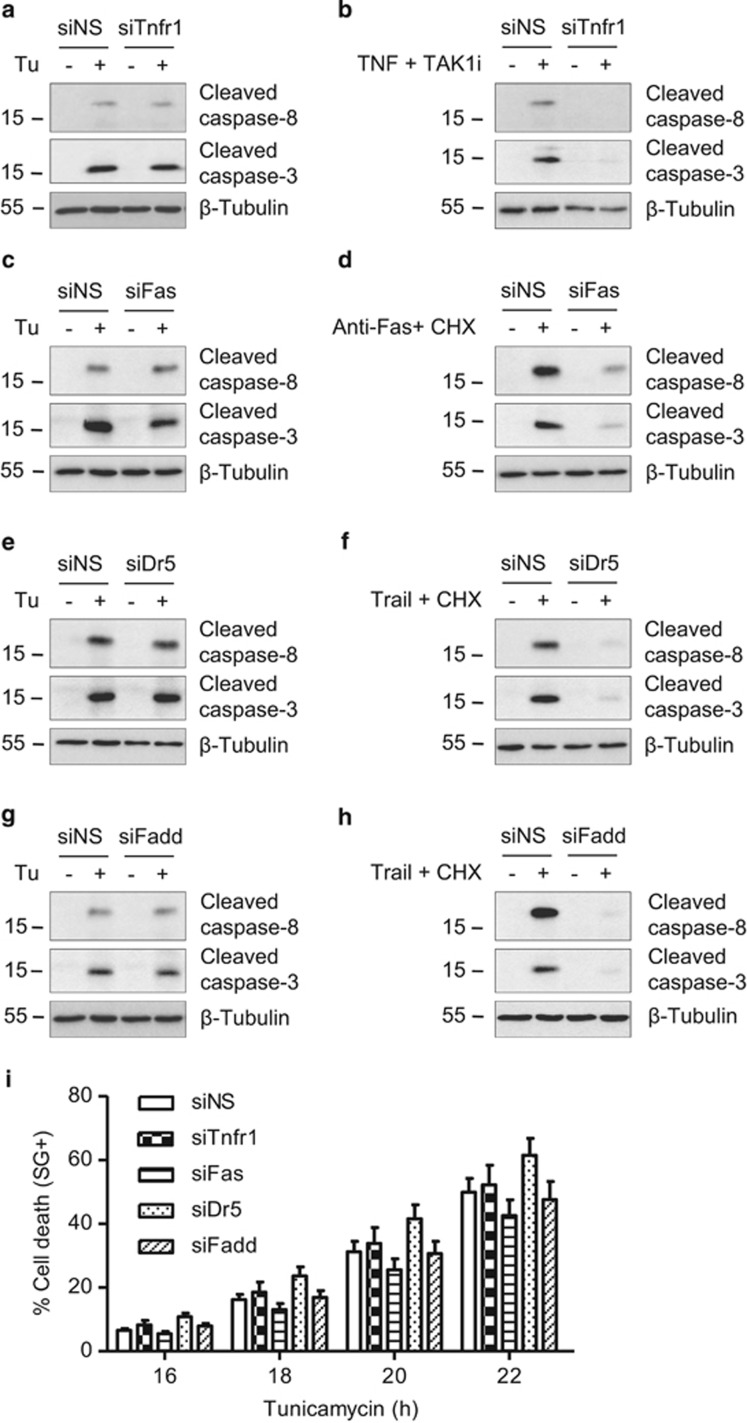
ER stress-induced caspase-8 activation occurs independently of the death receptors TNFR1, FAS, or DR5. (**a**–**h**) *Ripk1*^*+/+*^ MEFs were transfected with a control non-silencing siRNA (siNS) or or with siRNA targeting Tnfr1 (siTnfr1), Fas (siFas), Dr5 (siDr5), or Fadd (siFadd), and then exposed to 1 *μ*g/ml Tu for 12 h (**a**, **c**, **e**, and **g**). As control to test the functionality of repression, the cells were stimulated with TNF in combination with TAK1 inhibitor (TAK1i) (**b**), or with anti-Fas antibody (**d**), or Trail (**f** and **h**) in combination with cycloheximide (CHX) (**d**, **f**, and **h**). The cells were then lysed and immunoblotted as indicated. (**i**) *Ripk1*^*+/+*^ MEFs were transfected as before, stimulated with 1 *μ*g/ml Tunicamycin, and then the percentage of cell death was measured in function of time using the Fluostar Omega fluorescence plate reader. Error bars represent S.E.M. of three independent experiments

We next investigated the possibility that caspase-8 activation could still occur through the engagement of death receptors of the TNFR superfamily by autocrine production of their respective ligands, as previously suggested.^24, 48^ We found that the processing of caspase-8 and -3 induced by tunicamycin was not prevented by the use of blocking antibodies targeting TNF or TRAIL, or by a recombinant mouse Fas:Fc chimera, although these approaches efficiently blocked activation of the caspases when the agonists were provided exogenously (Figures 4a–f). Because ligand-independent TNFR1 as well as death receptor 5 (DR5) signaling have been reported during ER stress conditions,^23, 49^ we additionally tested the effect of TNFR1, FAS, and DR5 depletion by siRNA. As shown in Figure 5, neither Tnfr1, Fas, nor Dr5 repression had any impact on tunicamycin-induced caspase-8 or -3 processing (Figures 5a, c, and e) or cell death (Figure 5i) although the respective knockdown of these death receptors completely blocked caspases processing triggered by exogenous agonists (Figures 5b, d, and f). Accordingly, knockdown of the death receptor adaptor FADD had no effect on tunicamycin-induced caspases activation or cell death (Figures 5g–i). These results argue against the involvement of the classical death receptors TNFR1, FAS, and DR5 in tunicamycin-induced apoptosis, and suggest that caspase-8 activation is triggered by another pathway.

### RIPK1 interacts with IRE1

**Figure 6:**
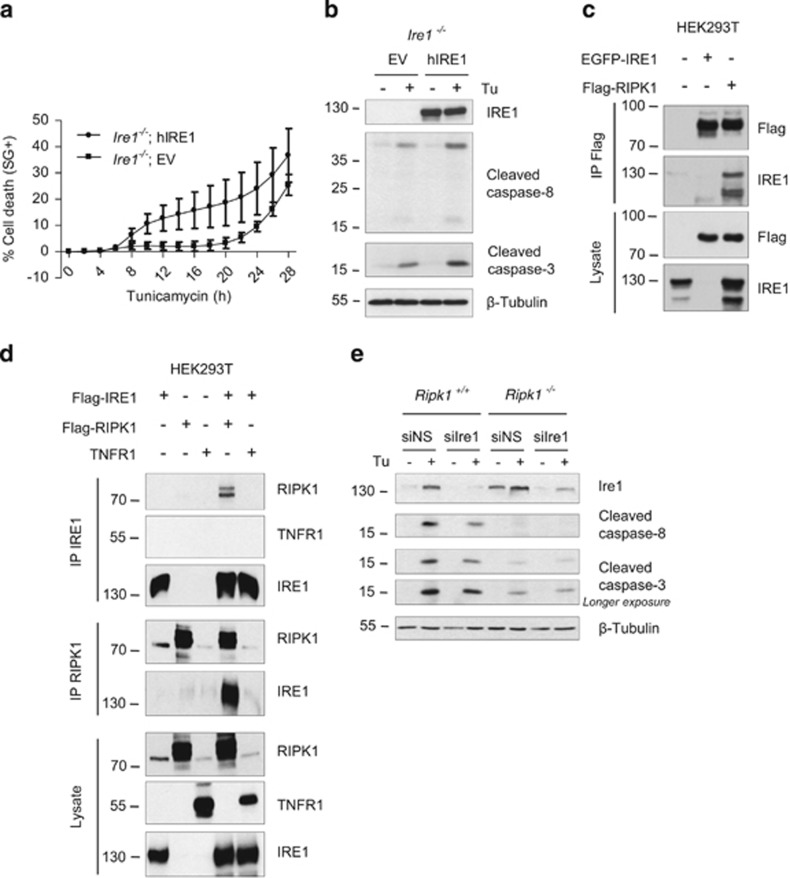
RIPK1 interacts with the pro-apoptotic receptor IRE1. (**a** and **b**) *Ire1*^*−/−*^ cells reconstituted with an empty vector (EV) or with a vector coding for hIRE1(hIRE1) were stimulated with 1 *μ*g/ml tunicamycin (Tu), and the percentage of cell death was measured in function of time using the Fluostar Omega fluorescence plate reader (**a**), or cell lysates obtained after 17 h of stimulation were immunoblotted as indicated (**b**). (**c**) HEK293T cells were transiently transfected with plasmids coding for EGFP-hIRE1 and/or Flag-hRIPK1, and RIPK1 was immunprecipitated (IP) using anti-Flag-coated beads. Cell lysates and immunoprecipitates were analyzed by immunoblot as indicated. (**d**) HEK293T cells were transiently transfected with plasmids coding for Flag-hIRE1, Flag-hRIPK1 and/or hTNFR1, and IRE1 (upper panels) or RIPK1 (middle panels) were immunoprecipitated with anti-IRE1 or anti-RIPK1 antibodies, respectively. Cell lysates and immunoprecipitates were analyzed by immunoblot as indicated. (**e**) *Ripk1*^*+/+*^ and *Ripk1*^*−/−*^ MEFs were transfected with a control non-silencing siRNA (siNS) or targeting Ire1 (siIre1) and then exposed to 1 *μ*g/ml Tu for 12 h. The cells were then lysed and immunoblotted as indicated

IRE1 can promote both survival and death pathways under ER stress conditions,^2, 3^ suggesting that the pro-apoptotic function of RIPK1 could be initiated by an interaction with IRE1. First, to confirm the pro-apoptotic role of IRE1 during ER stress, we analyzed cell death and processing of caspase-8 and -3 in response to tunicamycin in Ire1^*−/−*^ cells reconstituted with an empty vector or with a vector coding for hIRE1.^50^ As shown in Figures 6a and b, cell death induction as well as cleavage of caspase-8 and -3 were more pronounced in the IRE1-reconstituted cells, supporting a role for IRE1 in signaling toward apoptosis in response to tunicamycin treatment. A previous report suggests that IRE1 forms a complex with TNFR1 and RIPK1 for regulating JNK activation in response to ER stress.^23^ Although our results indicate that RIPK1 is not crucial for JNK activation, we still examined the interaction of RIPK1 with IRE1. To do so, we performed co-immunoprecipitation experiments in lysates of HEK293T cells ectopically expressing IRE1, RIPK1, and TNFR1. Interestingly, we confirmed interaction between RIPK1 and IRE1 when immunoprecipitating either IRE1 or RIPK1 (Figures 6c and d). However, no interaction between IRE1 and TNFR1 was detected when immunoprecipitating IRE1 (Figure 6d). Hence, our results confirm the previously reported interaction between IRE1 and RIPK1, and suggest that this interaction, which occurs independently of TNFR1, could contribute to the pro-apoptotic role of RIPK1 and IRE1. Accordingly, we observed that the knockdown of Ire1 decreased tunicamycin-induced caspases processing in *Ripk1*^*+/+*^ MEFs while it did not further protect MEFs already deficient for Ripk1 (Figure 6e).

## Discussion

So far, how the UPR switches from a pro-survival to a pro-death mode, as well as how it proceeds to the execution phase, are still incompletely understood. On the basis a previous report suggesting the requirement of RIPK1 for JNK activation by IRE1,^23^ we investigated the role of RIPK1 in ER stress-mediated death. Our data identified RIPK1 as a new mediator of ER stress-induced apoptosis that regulates the activation of caspase-8.

It is widely accepted that the UPR triggers the intrinsic pathway of apoptosis by regulating the expression and activation of Bcl-2 family members.^2, 3, 7^ However, some recent reports support the idea that ER stress can also trigger activation of the extrinsic apoptotic pathway through autocrine production of TNF *via* IRE1-mediated activation of NF-*κ*B,^24^ or through the upregulation of the TRAIL receptor DR5 by the PERK-CHOP pathway.^49, 51, 52, 53, 54^ In addition, ligand-independent signaling downstream of TNFR1 and DR5 has also been reported to trigger ER stress-mediated death.^23, 49, 51^ Accordingly, caspase-8 activation has been observed under ER stress conditions.^55, 56, 57^ Our results show that caspase-8 has a primarily role in ER stress-mediated apoptosis. These data therefore strongly suggest that activation of caspase-8 represents an early event under ER stress conditions, and that activation of the intrinsic mitochondrial pathway is a secondary event relying, for instance, on the cleavage of Bid by caspase-8.^58^ Although caspase-2 has previously been reported to mediate Bid cleavage during ER stress-mediated death,^15, 16, 59^ we did not detect any caspase-2 processing in our cellular model, which goes in line with a recent report questioning the requirement of caspase-2 for ER stress-induced death.^60^ Importantly, our results also demonstrate that caspase-8 activation in MEFs does not result from activation of the extrinsic pathway downstream of TNFR1, Fas, or DR5, highlighting the potential cell-specific characteristic of cell death signaling under unresolved ER stress conditions.

In agreement, we did not detect the formation of a complex containing RIPK1 and caspase-8, a prerequisite for RIPK1-dependent apoptosis downstream of TNFR1.^29, 33, 35, 61^ Moreover, cIAP1/2 depletion had no effect on ER stress-mediated death in our cellular model whereas they have a crucial role in TNFR-mediated cell death.^28, 29, 34, 35^ A BAP31-containing complex has been reported to function as a caspase-8-activating platform transducing apoptotic signals from the ER to the mitochondria under ER stress.^62, 63^ However, our preliminary results indicate that Bap31 repression in MEFs did not prevent tunicamycin-induced caspase-8 activation (data not shown). Further investigations are therefore required to fully understand the mode of activation of caspase-8 in response to ER stress and the involvement of RIPK1 in this process.

Several studies have previously reported a pro-apoptotic role of IRE1 under prolonged ER stress conditions.^11, 16, 17, 20, 24^ Our results using the *Ire1*^*−/−*^-reconstituted cells, and Ire1 knockdown experiments, confirm a pro-apoptotic function of IRE1. However, it is important to note that Ire1 deficiency only slightly protected the cells from tunicamycin-induced caspase activation in our cellular model. This can potentially be explained by the dual function of IRE1 that can mediate death but also survival pathways.^2, 3^ To favor protection from death, the death signaling branches emerging from IRE1 should be specifically targeted. For instance, we found that JNK inhibition provides a better protection toward caspase activation than Ire1 depletion. Similarly, we observed that Ripk1 depletion protects the cells from tunicamycin-induced apoptosis much more potently than Ire1 depletion. One possible interpretation is that Ripk1 depletion specifically affects the main killing branch emerging from IRE1. In line with this, we confirmed an interaction between IRE1 and RIPK1,^23^ indicating that RIPK1 can transmit signals initiated at the receptor. However, in contrast to the report of Yang *et al.*,^23^ our results indicate that RIPK1 is not essential for JNK activation, as measured by c-Jun phosphorylation. Although unlikely, we can at this stage not formally exclude an eventual difference in the transcriptional activity of AP1 between WT and Ripk1-deficient cells (a similar remark can also be made for CHOP). We also ruled out the possibility that RIPK1 regulates the survival function of IRE1 that depends on XBP-1 mRNA splicing. Since IRE1 recruits numerous proteins to its cytosolic domain to form a signaling molecular platform referred as the UPRosome,^64^ it is tempting to speculate that the binding of RIPK1 to IRE1 could modulate the kinetic and the amplitude of IRE1 response to ER stress by regulating the assembling and/or the composition of the UPRosome. Indeed, alteration of IRE1 signaling termination leads to excessive RIDD activity, which is proposed to favor cell death *versus* survival during prolonged ER stress.^11, 12, 13, 14, 16^ Further work is required to clearly characterize the consequences of the interaction between RIPK1 and IRE1. Indeed, we cannot exclude the possibility that the role of RIPK1 in caspase-8-mediated death goes beyond its interaction with IRE1.

In conclusion, our findings uncover a novel pro-apoptotic function of RIPK1 in conditions of unresolved ER stress. We demonstrate that RIPK1 controls death receptor-independent activation of caspase-8, which is crucial for apoptosis induction.

## Materials and Methods

### Cell culture and plasmids

MEFs and HEK293T cells were cultured in high glucose Dulbecco's modified Eagle's medium (DMEM) supplemented with 10% fetal calf serum and L-glutamine (200 mM). *Ripk1*^*+/+*^ and *Ripk1*^*−/−*^ MEFs have been reported previously.^33^ In brief, they were isolated from E12.5 littermate embryos and immortalized with SV40 large T antigen. Reconstitution of the *Ripk1*^*−/−*^ MEFs was done by lentiviral transduction. Viral particles were produced by transfecting HEK293T cells using calcium phosphate with a pLenti6-puro-HA-Ripk1 or pLenti6-puro-HA-Luciferase as a control together with the lentiviral packaging vectors pMD2-VSVG and pCMV-ΔR8.9.1. The medium was changed after 6 h, and the virus-containing supernatant was collected after 24 and 48 h and used to infect the *Ripk1*^*−/−*^ cells. Infected MEFs were then selected for 3 days by adding 2.5 *μ*g/ml puromycin to the medium. *Ire1*^*−/−*^ cells transduced with an empty pBabe vector or with a pBabe vector encoding FLAG- hIRE1 were kindly provided by David Ron (University of Cambridge, UK).^50^ These cells were cultured in DMEM supplemented with 10% fetal calf serum, L-glutamine, non-essential amino acids, and puromycin (3 *μ*g/ml). The EGFP-hIRE1 construct was a kind gift of Sarah Gerlo (University of Ghent). The Flag-hIRE1 plasmid has previously been reported,^65^ and the Flag-RIPK1 construct is a kind gift from Z Chen (University of Texas Southwestern).

### Reagents and antibodies

Tunicamycin was obtained from Sigma-Aldrich (St. Louis, MO, USA; #T7765) and used at 1 *μ*g/ml. Necrostatin-1 (Calbiochem, Merck KGaA, Darmstadt, Germany; #480065) was used at 10 *μ*M. Z-VAD-fmk (Bachem, Bubendorf, Switzerland; #N-1510) was used at 20 *μ*M. Doxycycline (Sigma-Aldrich #D9891) was used at 1 *μ*g/ml. SP600125 JNK inhibitor (Calbiochem #420119) was used at 10 and 20 *μ*M. TAK1 kinase inhibitor (AnalytiCon Discovery GmbH, Potsdam, Germany; #NP-0009245) was used at 1 *μ*M. Recombinant human TNF was produced in our laboratory, purified to at least 99% homogeneity, had a specific biological activity of 3–107  IU/mg, and was used at 600 IU/ml (20 ng/ml). Cycloheximide was from Sigma-Aldrich (#C7698). TRAIL (SuperKiller) was from Enzo Life Sciences (Farmingdale, NY, USA; #ALX-201-130-C020) and was used at 100 ng/ml. Recombinant mouse Fas:Fc chimera was from R&D Systems (Abingdon, UK; #435-FA). Antibodies were purchased from the following companies: anti-RIPK1 (BD Biosciences, San Jose, CA, USA; #610459), anti-caspase-8 (Abnova, Jhongli, Taiwan; #MAB3429), anti-cleaved caspase-8 (Cell Signaling, Danvers, MA, USA; #9429), anti-caspase-3 (Cell Signaling #9662), anti-cleaved caspase-3 (Cell Signaling #9661), anti-*β*-tubulin (Abcam, Cambridge, UK; #ab6046-200), anti-cleaved PARP (Asp214) (Cell Signaling #9544S), anti-phospho-c-Jun (Cell Signaling #2361S), anti-c-Jun (Santa Cruz Biotechnology, Dallas, TX, USA; #sc-1694), anti-IRE1 (Cell Signaling #3294), anti-CHOP (Cell Signaling #2895S), anti-caspase-9 (Cell Signaling #9508), anti-Fas (BD Biosciences #554254), blocking anti-TRAIL (eBiosciences, San Diego, CA, USA; #165951), anti-Flag M2 (Sigma-Aldrich #A2220), anti-IRE1 used for immunoprecipitation (Santa Cruz Biotechnology #sc-20790), and anti-TNFR1 (clone H5) (Santa Cruz Biotechnology #sc-8436). Blocking anti-TNF was from Bioceros B.V (Utrecht, The Netherlands; #XT-22).

### Analysis of cell death and caspase-3 activity

Cell death and caspase-3 like activity analysis using the Fluostar Omega fluorescent plate reader (BMG Labtech GmbH, Ortenberg, Germany) was performed as previously described.^33^ Briefly, the cells were seeded in triplicate at 7500 cells/well in a 96-well plate. The next day the cells were treated with the indicated compounds in the presence of 5 *μ*M SYTOX Green (SG) (Life Technologies, Carlsbad, CA, USA) and for some experiments in combination with 20 *μ*M DEVD-AMC (PeptaNova GmbH, Sandhausen, Germany). SG and AMC fluorescence intensity were measured in function of time with excitation/emission filters of 485/520 nm and 360/460 nm, respectively. The percentage of cell death was calculated as (induced SG fluorescence−background SG fluorescence)/(maximal SG fluorescence−background SG fluorescence)X100. The maximal SG fluorescence was obtained by full permeabilization of the cells using 0.05% Triton X-100. Caspase-3like activity was calculated as (induced AMC fluorescence−background AMC fluorescence).

### RT-qPCR

*Ripk1*^+/+^ and *Ripk1*^*−/−*^ MEFs were incubated with 1 *μ*g/ml tunicamycin for the indicated times, and total RNA was isolated from cell lysates by using the RNeasy plus mini kit (Qiagen, Hilden, Germany). CHOP mRNA as well as the housekeeping gene HPRT were examined by RT-qPCR.

### Immunoprecipitation

After overnight transfection in HEK293T, or after tunicamycin treatment in MEFs, cells were washed with PBS and then lysed in cold lysis buffer (10 mM Tris-HCl (pH 7.5), 150 mM NaCl, 1% NP-40 and 10% glycerol), supplemented with EDTA-free protease inhibitor cocktail tablets (Roche Diagnostics, Basel, Switzerland) and phosphatase inhibitor cocktail tablets (Roche Diagnostics). Flag-RIPK1 was immunoprecipitated using anti-Flag M2 affinity gel (Sigma-Aldrich). For endogenous caspase-8 immunoprecipitation, cells were treated with tunicamycin in the presence of 20 *μ*M Z-VAD, and then caspase-8 was immunoprecipitated using home-made anti-caspase-8 antibody. Anti-caspase-8, anti-IRE1, and anti-RIPK1 antibodies were coupled to protein A beads for immunoprecipitation. All immunoprecipitations were performed overnight at 4 °C. The beads were then recovered by centrifugation, and immunoprecipitates were washed three times in cold lysis buffer, and eluted in Laemmli's buffer. Immunoprecipitates were then analyzed by immunoblot.

### RNA interference

For shRNA-based knockdown, Mission shRNA control or caspase-8 (TRCN0000012243, Sigma-Aldrich) plKO.1 puro vectors were transfected together with the lentiviral packaging vectors pMD2-VSVG and pCMV-ΔR8.9.1 using the standard calcium phosphate method. The medium was changed after 6 h, and the sample was collected 48 h post transfection. The virus-containing supernatants were then used to infect MEF cells, which were then selected for 4 days by adding 3 *μ*g/ml puromycin to the medium. For RNAi-based knockdown, synthetic non-silencing (D-001810-01) and SMARTpool mouse Tnfr1 (L-060201-01), Fas (L-045283-00), Dr5 (L-050949-00), Fadd (L-040488-00), and Ire1 (L-041030-00) siRNA were purchased from Dharmacon (Thermo Fisher Scientific, Waltham, MA, USA). The cells were seeded in 6-well plate, and 25 nM siRNA was transfected using DharmaFECT 1 reagent (Dharmacon, Thermo Fisher Scientific) according to the manufacturer's recommendations. After 48 h of transfection, the cells were used for experiments.

### Statistics

Statistical analysis were performed by two-way ANOVA and Bonferroni post-tests. A *P*<0.01 was considered as significant.

## Abbreviations

ER: endoplasmic reticulum
UPR: unfolded protein response
RIPK1: receptor-interacting serine/threonine protein kinase 1
cIAP1/2: cellular inhibitor of apoptosis 1 and 2
MEF: mouse embryonic fibroblast
PARP: poly [ADP-ribose] polymerase
IRE1: inositol-requiring protein 1
PERK: protein kinase RNA-like ER kinase
ATF6: activating transcription factor 6
CHOP: C/EBP-homologous protein
ERO1*α*: ER oxidase 1*α*
IP3: inositol 1,4,5-trisphosphate
XBP-1: X-box-binding protein 1
RIDD: regulated IRE1-dependent decay
JNK: c-Jun N-terminal kinase
TNFR: tumor necrosis factor receptor
TRAF2: (TNFR)-associated factor 2
ASK1: apoptosis signal-regulated kinase 1
TAK1: transforming growth factor-beta-activated kinase 1
MAPK: mitogen-activated protein kinase
NF-*κ*B: nuclear factor-kappa-B
FADD: FAS-associated death domain protein
Nec1: necrostatin-1
IL-1*β*: interleukin-1 beta
TRAIL: TNF-related apoptosis-inducing ligand
I*κ*B*α*: NF-kappa-B inhibitor alpha
HEK: human embryonic kidney
DR5: death receptor 5
