# Supplementary material for: RIPK1 promotes death receptor-independent caspase-8-mediated apoptosis under unresolved ER stress conditions
Source: Cell Death Dis. 2014 Dec 4;5(12):e1555–. doi: 10.1038/cddis.2014.523 (PMC4649839; doi:10.1038/cddis.2014.523)
Supplement: Supplementary Information [file cddis2014523x1.pdf]

Supplemental information

Materials and Methods:

Cell lines, reagents and antibodies:

Wild type (A3) and *caspase-8*$^{-/-}$ (I9.2) Jurkat cells were cultured in RPMI 1640 medium (Life Technologies) supplemented with 10% fetal calf serum and L-glutamine (200 mM). thapsigargin (#T9033) and brefeldin A (#B7651) were purchased from Sigma Aldrich. Compound BV6 (Genentec, Inc) was used at 500 nM. Recombinant Il-1β is produced in our laboratory. The anti-cIAP1 (#ALX-803-335-C100) was purchase from Enzo life Sciences, the anti-spliced Xbp-1 (#619501) from BioLegend, the anti-caspase-8 (clone B9-2) (#556466) from BD Pharmingen, the anti-IκBα (#sc-371) from Santa Cruz Biotechnology, the anti-cleaved caspase-3 (#9661) and anti-PARP (clone 46D11)(#9532) from Cell Signaling Technology.

Cell death analysis using the high content bioimager BD PathwayTM 855 instrument

Cell death analysis using the high content bioimager BD PathwayTM 855 instrument (BD Biosciences) was performed as previously described [1]. Briefly, cells were seeded in triplicate at the density of 7500 cells per well in 96-well BD-imaging plates. The day after, the cells were treated with the indicated compounds in the presence of 2 µg/ml Hoechst 33342 and 1 µg/ml propidium iodide (PI). Hoechst labeling was used to segment the nuclei and to extract Hoechst and PI intensity values of each nucleus, with BD Attovision analysis software. The percentage of dead cells was calculated in function of time as the percentage of nuclei with PI fluorescence intensity above the threshold of nuclei PI fluorescence of untreated cells.

RT-PCR

Total RNA was extracted and purified with the RNeasy plus mini kit (Qiagen). cDNA was amplified using GoTaq® Green Master Mix (Promega) and the following primers for mouse Xbp-1: 5’-GAACCAGGAGTTAAGAACACG-3’ (Forward) and 5’-AGGCAACAGTGTCAGAGTCC-3’ (Reverse). A PCR amplification cycle included denaturation at 95°C for 5 min, followed by 35 cycles at 95°C for 1 min, 58°C for 30 sec and 72°C for 30 sec, and a final extension at 72°C for 5 min.

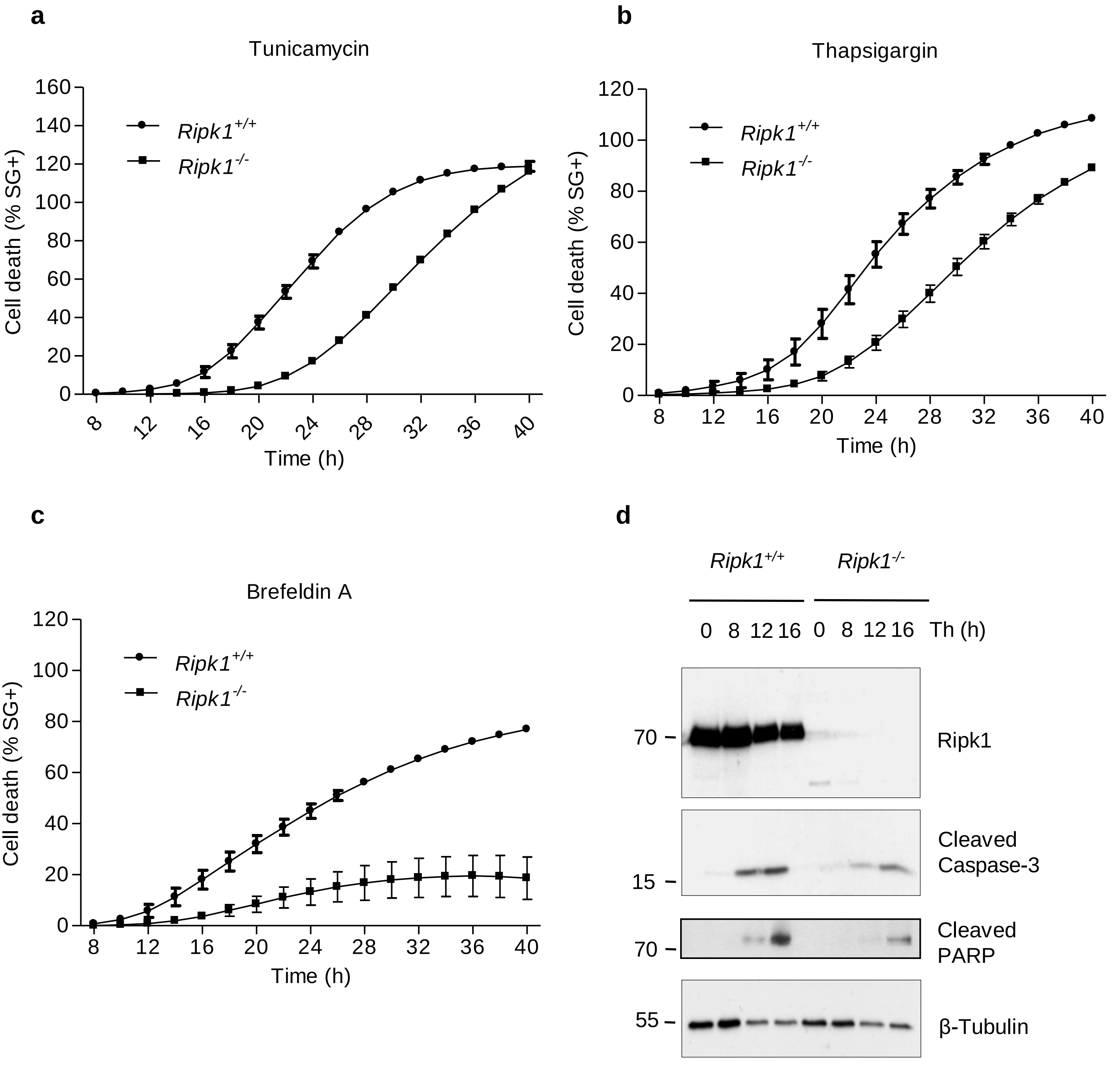

Figure S1: Ripk1 deficient cells are protected against death induced by various ER stress inducers. (**a-c**) *Ripk1*$^{+/+}$ and *Ripk1*$^{-/-}$ MEFs were exposed to 1µg/ml tunicamycin, 0.1 µM thapsigargin, or 0.5 µM brefeldin and the percentage of cell death was measured in function of time using the Fluostar Omega fluorescence plate reader. Error bars represent S.E.M. of two independent experiments (**d**) *Ripk1*$^{+/+}$ and *Ripk1*$^{-/-}$ MEFs were treated with 0.1 µM thapsigargin for the indicated time, and then lysed and immunoblotted as indicated.

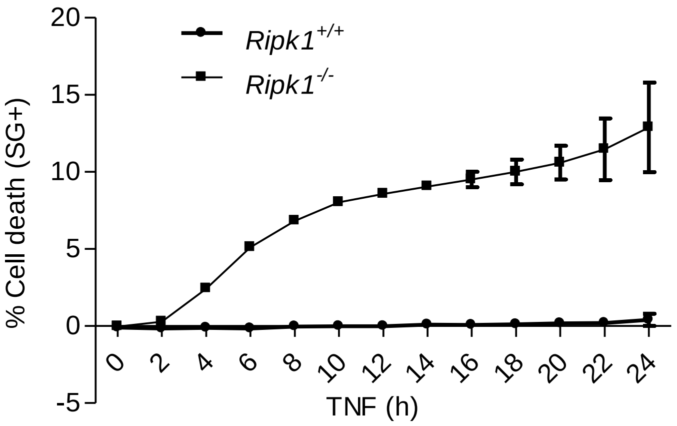

Figure S2: Ripk1 deficient cells are sensitized to TNF-induced death. *Ripk1*$^{+/+}$ and *Ripk1*$^{-/-}$ MEFs were stimulated with TNF, and the percentage of cell death was measured in function of time using the Fluostar Omega fluorescence plate reader. Error bars indicate the standard deviation from triplicate samples. The result is representative of two independent experiments.

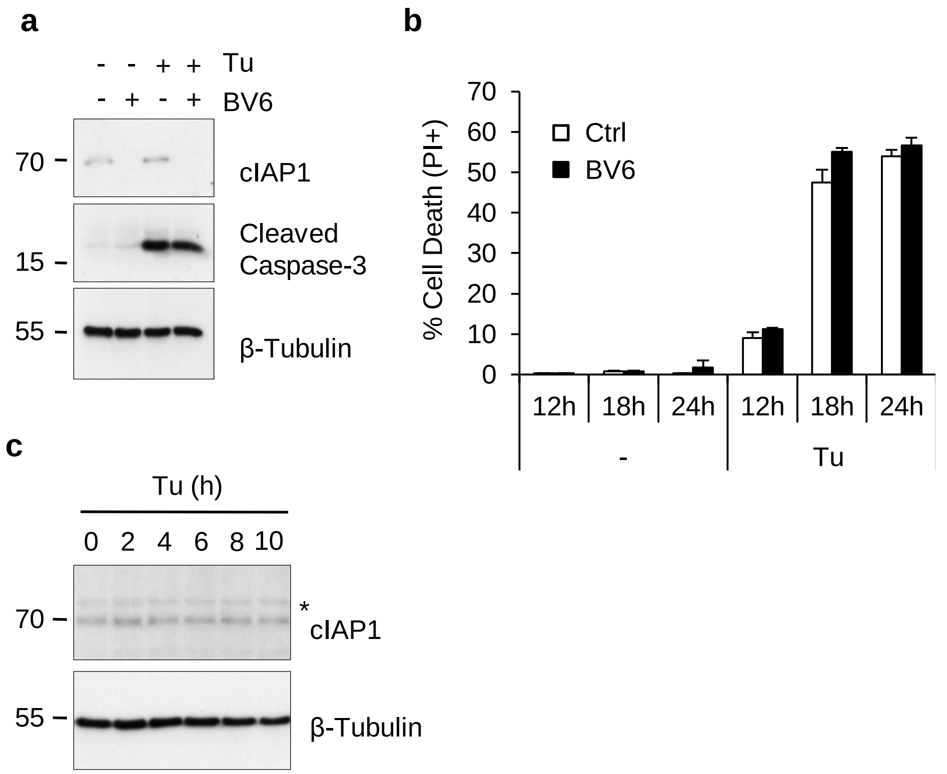

Figure S3: cIAP1/2 do not regulate tunicamycin (Tu)-induced death. (**a**) *Ripk1*$^{+/+}$ MEFs were incubated for 30 min with BV6, and then stimulated with 1 µg/ml Tu for 12h. The cells were then lysed and immunoblotted as indicated. (**b**) *Ripk1*$^{+/+}$ MEFs were incubated for 30 min with BV6, and then stimulated with 1 µg/ml Tu. The percentage of cell death (PI fluorescence) was measured in function of time using the high content bioimager BD Pathway™ 855 instrument. Error bars indicate the standard deviation from triplicate samples. The result is representative of two independent experiments. (**c**) *Ripk1*$^{+/+}$ MEFs were exposed to 1 µg/ml Tu for the indicated time, and then lysed and immunoblotted as indicated. The asterisk indicates a non-specific band.

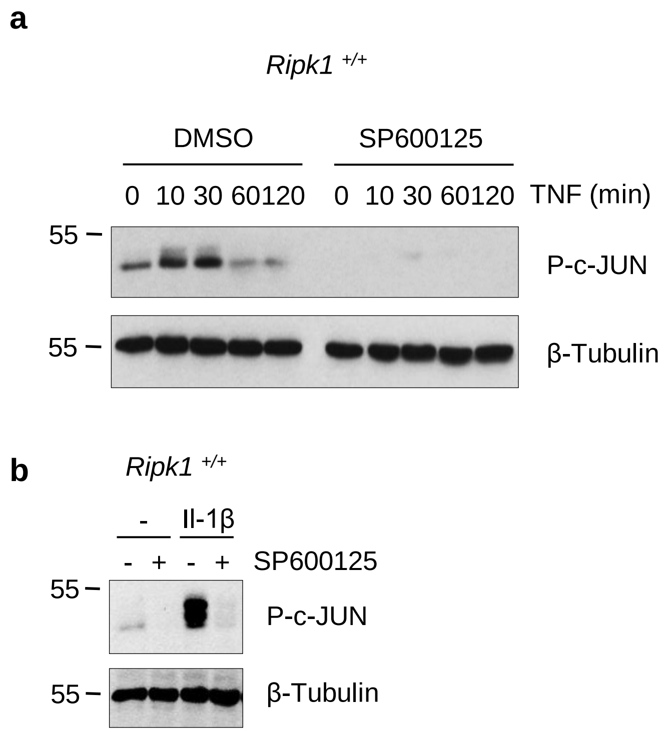

Figure S4: The JNK inhibitor SP600125 efficiently blocks TNF- and Il-1β-induced phosphorylation of c-Jun. (**a-b**) *Ripk1*$^{+/+}$ MEFs were incubated for 30 min with 20 µM SP 600125, and then stimulated with TNF (**a**), or with 10 ng/ml Il-1β for 10 min (**b**). Cell lysates were immunoblotted as indicated.

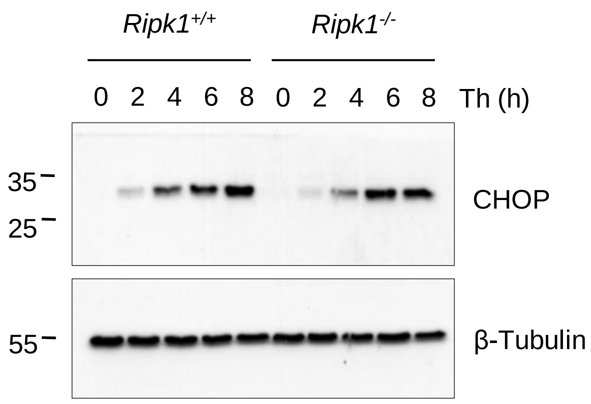

Figure S5: Ripk1 deficiency does not alter thapsigargin-induced CHOP expression. *Ripk1*$^{+/+}$ and *Ripk1*$^{-/-}$ MEFs were exposed to 0.1 µM thapsigargin (Th) for an increasing period of time, and then lysed and immunoblotted as indicated.

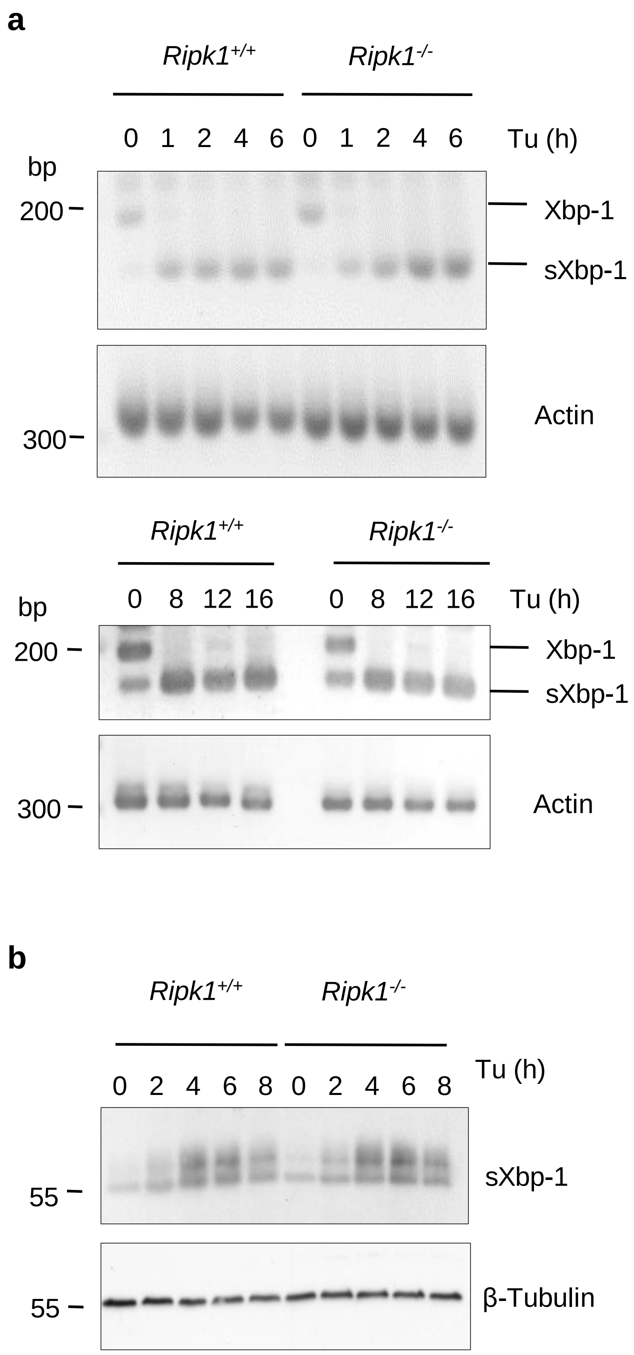

Figure S6: Ripk1 deficiency does not alter tunicamycin-induced Xbp-1 mRNA splicing or spliced Xbp1 protein expression. (**a-b**) *Ripk1*$^{+/+}$ and *Ripk1*$^{-/-}$ MEFs were exposed to 1 µg/ml tunicamycin (Tu) for the indicated time, and then Xbp-1 mRNA splicing was analyzed by RT-PCR (**a**) and spliced Xbp-1 (sXbp-1) protein expression was analyzed by immunoblot (**b**).

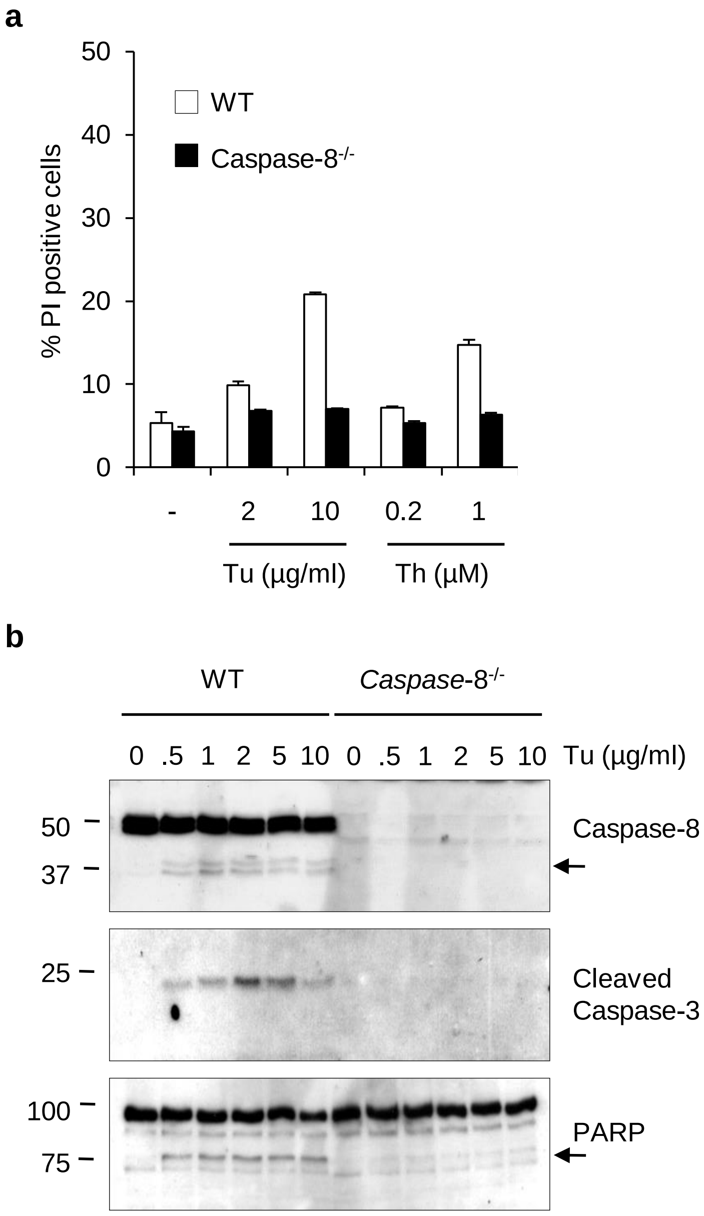

Figure S7: Caspase-8 deficient Jurkat cells are protected against ER stress-induced apoptosis. (**a**-**b**) Wild type (WT) JA3 and caspase-8 deficient (*caspase-8*$^{-/-}$) I9.2 Jurkat cells were treated with various concentrations of tunicamycin (Tu) or thapsigargin (Th). (**a**) After 24h stimulation the percentage of cell death (PI fluorescence) was measured by flow cytometry. Error bars indicate the standard deviation from triplicate samples. (**b**) After 16h stimulation, the cells were lysed and immunoblotted as indicated. Arrows indicated cleaved fragment of proteins.

Bibliography:

1. Duprez L, Takahashi N, Van Hauwermeiren F, Vandendriessche B, Goossens V, Vanden Berghe T, *et al.* RIP kinase-dependent necrosis drives lethal systemic inflammatory response syndrome. *Immunity* 2011 Dec 23; **35**(6)**:** 908-918.
